# ORTEGA v1.0: an open-source Python package for context-aware interaction analysis using movement data

**DOI:** 10.1186/s40462-024-00460-2

**Published:** 2024-03-09

**Authors:** Rongxiang Su, Yifei Liu, Somayeh Dodge

**Affiliations:** Department of Geography, University of California Santa Barbara, Santa Barbara, 93106 USA

**Keywords:** Animal interaction, GPS tracking data, Interaction analysis, Movement analysis, Movement data, Potential path area, Time geography, Telemetry data

## Abstract

**Background:**

Interaction analysis via movement in space and time contributes to understanding social relationships among individuals and their dynamics in ecological systems. While there is an exciting growth in research in computational methods for interaction analysis using movement data, there remain challenges regarding reproducibility and replicability of the existing approaches. The current movement interaction analysis tools are often less accessible or tested for broader use in ecological research.

**Results:**

To address these challenges, this paper presents ORTEGA, an Object-oRiented TimE-Geographic Analytical tool, as an open-source Python package for analyzing potential interactions between pairs of moving entities based on the observation of their movement. ORTEGA is developed based on one of the newly emerged time-geographic approaches for quantifying space-time interaction patterns among animals. A case study is presented to demonstrate and evaluate the functionalities of ORTEGA in tracing dynamic interaction patterns in animal movement data. Besides making the analytical code and data freely available to the community, the developed package also offers an extension of the existing theoretical development of ORTEGA for incorporating a context-aware ability to inform interaction analysis.

**Conclusions:**

ORTEGA contributes two significant capabilities: (1) the functions to identify potential interactions (e.g., encounters, concurrent interactions, delayed interactions) from movement data of two or more entities using a time-geographic-based approach; and (2) the capacity to compute attributes of potential interaction events including start time, end time, interaction duration, and difference in movement parameters such as speed and moving direction, and also contextualize the identified potential interaction events.

## Background

Increasing access to growing repositories of animal tracking data (e.g. Movebank [1, 2]) has created unprecedented opportunities to advance the science of movement ecology and substantially contributed to our understanding of animal behaviors of animals [3, 4]. While our movement data collection has become ubiquitous, methods to analyze and make sense of these complex data sets are not broadly available to researchers. For example, intuitive open-source tools to analyze and visualize the dynamic interactions or contacts between moving entities are lacking [5–7]. This study develops and assesses a new open-source Python package to analyze and map space-time interactions between two or more moving entities. Python has become the most used programming language for spatial data analysis and has greater development capabilities than other programming languages such as R, especially for large data sets. In this research, the term *interaction* refers to potential contacts between individuals in space and time. Such contact does not necessarily indicate the occurrence of physical or social interaction, but it may lead to exposure to risk factors or opportunities for social interaction.

Dynamic interaction [8, 9] between a pair of moving entities can be an *encounter* (i.e., a brief contact in space and time), or it can be either a *concurrent interaction* (synchronous movement in proximity over a certain time interval) or *delayed interaction* (or indirect/asynchronous, i.e., visiting the same location with a time lag). Traditional techniques to quantify dynamic interactions primarily rely on the proximity between two moving entities, often determined by user-defined spatial and temporal thresholds [8, 10, 11]. However, the effectiveness of the proximity-based approaches decreases when interacting individuals’ paths are not tracked simultaneously due to varied sampling rates, signal loss or imperfect tracking, or when the interactions are delayed (e.g., two animals visit the same location at different times) [4, 9]. In contrast, the time-geographic-based approaches provide a more robust framework to identify potential encounters as well as concurrent and delayed interactions between individuals [6, 9, 12–15]. This is mainly because the time-geographic-based approaches incorporate the uncertainty of positioning and gaps in movement data by considering potential locations accessible to moving individuals between consecutive tracking points.

ORTEGA (Object-oRiented TimE-Geographic Analytical approach) is an emerging time-geographic-based approach which can be used to identify various types of interactions and their duration at a reasonable computation cost, as we demonstrate later in the “Results” section [6, 9, 15]. This paper implements and evaluates a new extension of the tool, ORTEGA version 1.0, as an open-source Python package^1^^,^^2^ for analyzing, contextualizing, and mapping interactions between entities based on their movement observations (i.e., movement tracking data). The Python package ORTEGA v1.0 is built on top of the existing theoretical developments of ORTEGA, originally introduced in [6], and further extended in [9]. The developed package also offers an extension of ORTEGA for incorporating context-aware capacities to inform interaction analysis. By incorporating information on the context of movement, the outcomes can help us better distinguish between meaningful (or potentially intentional) and non-meaningful (or incidental) interactions.

Currently, there is a limited number of tools that offers time geographic-based analytical functions for interaction analysis. One example is PySTPrism, a toolbox integrated into ArcGIS Pro Desktop designed for voxel-based space-time prisms modeling [16]. Additionally, wildlifeDI [7] and STPtrajectories^3^ are two R packages capable of calculating and evaluating time-geographic elements. However, these tools are either rather limited in full open-source accessibility or may not be specifically tailored for utilizing time geography in the analysis of potential interactions based on movement tracking data. The ORTEGA v1.0 package contributes two significant capabilities: (1) the functions to identify potential interactions (e.g., brief encounters, concurrent interactions, delayed interactions) from movement data of two or more entities using a time-geographic-based approach; and (2) the capacity to compute attributes of potential interaction events including start time, end time, interaction duration, and difference in movement parameters such as speed and moving direction, and also contextualize the identified potential interaction events. To the best of our knowledge, the present ORTEGA is the first open-source Python package that offers a time-geographic-based interaction analytical tool for movement ecology research. To advance the reproducibility and replicability of this research, the developed package makes ORTEGA and its new extensions along with an example data set accessible to the community. To demonstrate the various functions implemented in ORTEGA v1.0, this paper presents case studies using long-term movement tracking data of two migratory turkey vultures [17] to analyze their dynamic interactions. For simplicity, this case study uses only two birds to demonstrate the application of ORTEGA’s functions and is not intended as comprehensive research. It is important to note that ORTEGA is capable of handling interaction analysis for a larger number of individuals. ORTEGA is built upon existing Python’s matplotlib [18], numpy [19], shapely [20], and pandas [21] libraries.

## Implementation

### Input data

ORTEGA v1.0 (from here on ORTEGA refers to ORTEGA version 1) accepts conventional movement tracking data of a set of moving individuals, such as GPS or telemetry data, in the form of comma-separated values (CSV) files. The movement data should at least include a unique identifier for each individual (or entity), a set of coordinates (geographic coordinates in floating-point format, provided in separate columns) representing the movement observations of each entity, and the corresponding timestamp of each observation (in Python “datetime” format).

### ORTEGA’s algorithm description

**Fig. 1:**
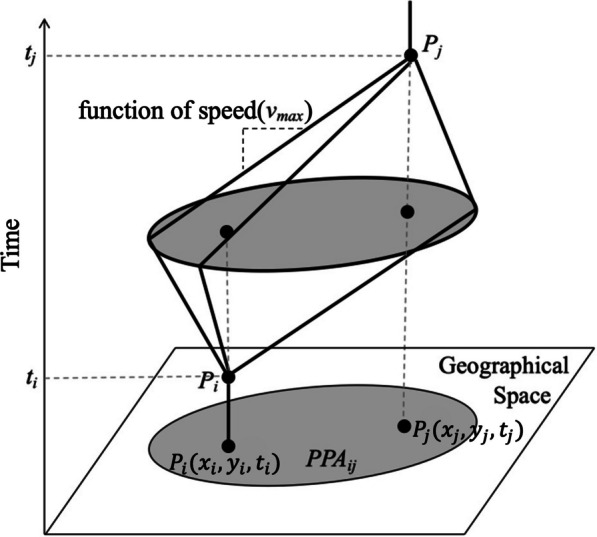
Illustration of the space–time prism and potential path area in a 3D space. $P_i(x_i,y_i,t_i)$ and $P_j(x_j,y_j,t_j)$ are the two consecutive tracking points. $v_{max}$ is the maximum speed capacity of the moving entity at the time interval $[t_i,t_j]$ given the two fixed locations $P_i$ and $P_j$. $PPA_{ij}$ denotes the potential path area in a 2D Euclidean space between $P_i$ and $P_j$ (modified from [25])

In time geography, the activity space of a moving entity can be measured by a space-time prism which is shaped by a pair of origin and destination locations (denoted by $P_i(x_i,y_i,t_i)$ and $P_j(x_j,y_j,t_j)$), a time budget ($t_j-t_i$), and the maximum speed capacity ($v_{max}$), as shown in Fig. 1 [22]. The projection of a space-time prism onto a two-dimensional Euclidean space is called the Potential Path Area (PPA), which delimits accessible locations that a moving entity can potentially reach given two fixed locations, a time budget and its maximum speed [23, 24]. Readers are referred to [25] for the mathematical definitions for various concepts in time geography. Using the time geography framework, the potential interactions between moving entities can be identified by intersecting their PPA ellipses along their trajectories [6, 9, 12–14].

**Fig. 2:**
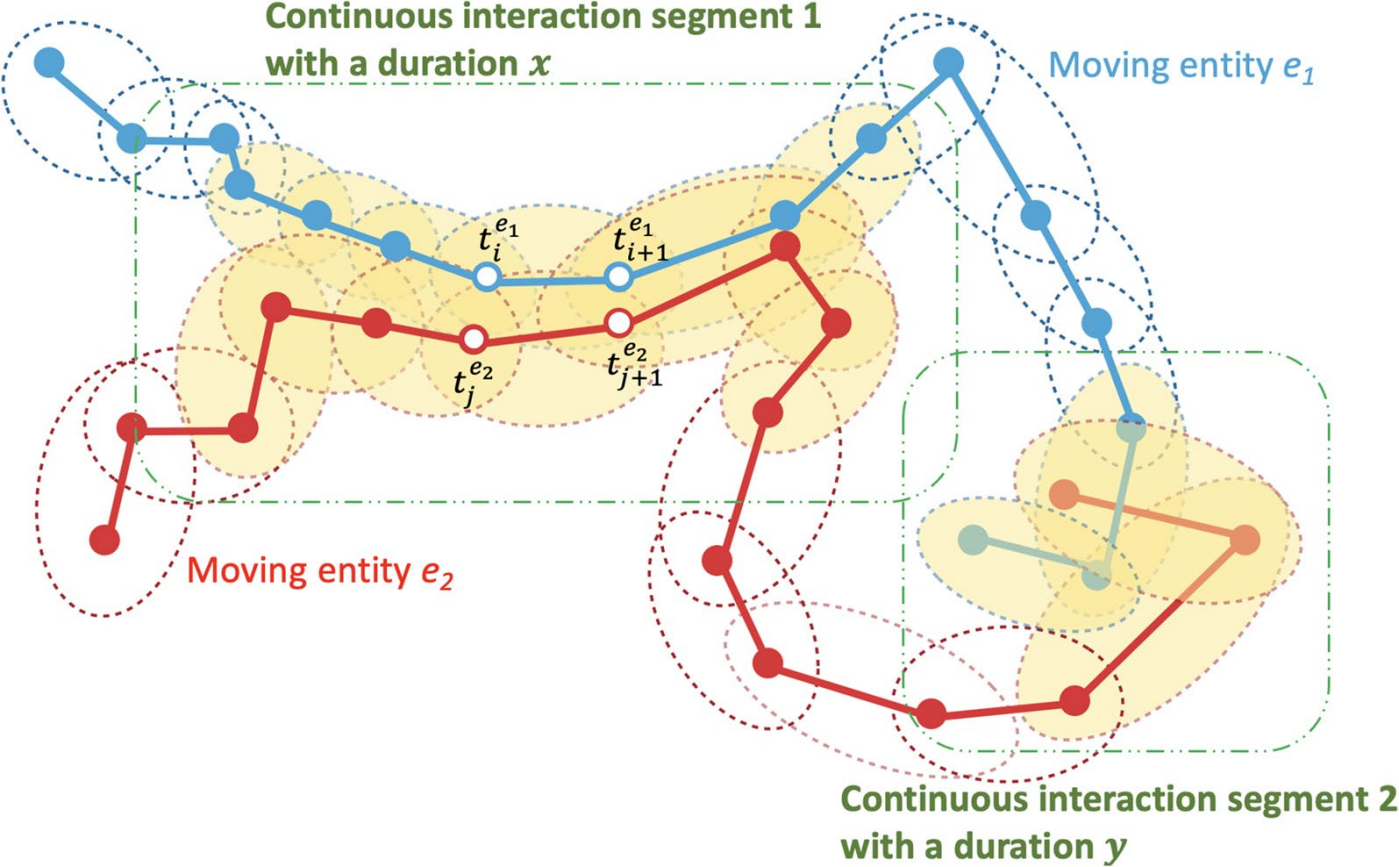
Illustration of ORTEGA for identifying potential interaction between two moving entities (modified from [9]). Yellow-filled ellipses represent intersecting PPAs where potential interaction can occur. For each pair of intersecting PPAs, the following condition should be met, $[t_i^{e_1}-\tau ,t_{i+1}^{e_1}+\tau ] \cap [t_j^{e_2},t_{j+1}^{e_2}] \ne \phi$ or $[t_i^{e_1},t_{i+1}^{e_1}] \cap [t_j^{e_2}-\tau ,t_{j+1}^{e_2}+\tau ] \ne \phi$, where $t_i^{e_1}, t_{i+1}^{e_1}, t_j^{e_2},t_{j+1}^{e_2}$ are the times when moving entities visit the corresponding GPS location. The two green dashed rectangles highlight two continuous interaction segments. The interaction duration can be computed by taking the difference between a continuous interaction segment’s start and end times. In Segment 1 of continuous interaction, the duration (*x*) exceeds the threshold value ($\delta _t$), classifying it as a concurrent interaction. In contrast, Segment 2, with a duration (*y*) shorter than $\delta _t$, is categorized as an encounter

As shown in Fig. 2, ORTEGA identifies potential interactions by first judging if two PPAs of the two moving entities intersect spatially, and whether their time intervals overlap within a predefined allowable short *time window* $\tau$. If so, a potential concurrent interaction is identified. The parameter $\tau$ is essential because, in reality, the movement tracking of two individuals is often unsynchronized or collected at different sampling rates. The parameter is domain-specific and usually can be the same as the average temporal resolution of the input movement data. It can also be set to zero for a strict synchronous alignment, especially when high-resolution data are available. To detect delayed interactions, the user needs to specify a *time interval* $[\tau ^a,\tau ^b]$ so that interactions that occur with a time lag falling within the specified range of $\tau ^a$ to $\tau ^b$ can be identified. Both $\tau ^a$ and $\tau ^b$ should be greater than $\tau$ and can be determined according to the study’s objectives. Additionally, by traversing every pair of intersecting PPAs based on their sequential order (e.g., yellow-filled ellipses in Fig. 2), we can extract continuous interaction segments (e.g., the portions highlighted with the green dashed rectangles) and compute their durations. The interaction duration is computed by taking the difference between a continuous interaction segment’s start and end times. Finally, if the duration of a continuous interaction segment is smaller than $\delta _t$, the interaction event is classified as an encounter. Otherwise, it is classified as a concurrent interaction of a duration equal to the difference between the start and end times of the continuous intersected PPA sequence. The concept of duration may not apply to delayed interactions, as individuals are present in the same space but at different times. The readers are referred to [6, 9] for further technical details of ORTEGA and the interaction analysis algorithms.

### Contextualizing interaction analysis

ORTEGA v1.0 extends its existing implementation [6] by incorporating functions for context-aware interaction analytics. Context is considered as the circumstance of movement or any internal or external variables influencing movement. The goal is to understand the relationships between identified potential interactions and the contexts when interactions occur. The fusion of large movement data and auxiliary environmental and behavioral variables can help us make sense of complex patterns captured in movement observations and better understand animal behavior. In the current version, the contextual correlates are modeled as attributes of the PPAs in ORTEGA’s object-oriented scheme. Examples of contextual attributes include behavioral states, land cover characteristics, vegetation, seasonality, temperature, etc. ORTEGA v1.0 supports as many numerical or categorical attributes as the user wishes to include. These attributes can directly be extracted from the original movement observations, if it is enriched with behavioral or environmental variables, or they can be computed from the coordinates, for example, movement parameters such as speed and direction. Software packages such as Env-DATA [26] can also be used to annotate movement observations with external environmental variables such as weather conditions, vegetation, etc. prior to interaction analysis.

### ORTEGA v1.0 Python package

ORTEGA v1.0 is written in Python language. Table 1 describes ORTEGA’s main functions. This section introduces the ORTEGA v1.0 Python package and provides step-by-step implementation instructions.

**Table 1:** Description of ORTEGA’s functions

Function	Description
interaction_analysis()	Identifies spatiotemporal intersecting PPAs after the user specifies the short time window (for identifying concurrent interaction) or time interval (for delayed interaction) during the initialization step; computes the difference in speeds and movement directions for each intersecting PPA pair; and extracts continuously intersecting PPA segments (i.e., interaction events) if any exists
compute_interaction_duration()	Computes the duration of each concurrent interaction event. This function only applies to concurrent interaction
compute_ppa_perimeter()	Descriptive statistics of PPA ellipse’s perimeter for each moving individual
﻿compute_ppa_area()	Descriptive statistics of PPA ellipse’s area for each moving individual
compute_ppa_interval()	Descriptive statistics of PPA time interval for each moving individual
compute_ppa_speed()	Descriptive statistics of PPA speed for each moving individual
attach_attributes()	Attaches attributes such as contextual variables to the dataframe of intersecting PPAs (derived from the function *interaction_analysis()*) for further correlation analysis

#### Setting up ORTEGA

**Fig. 3:**
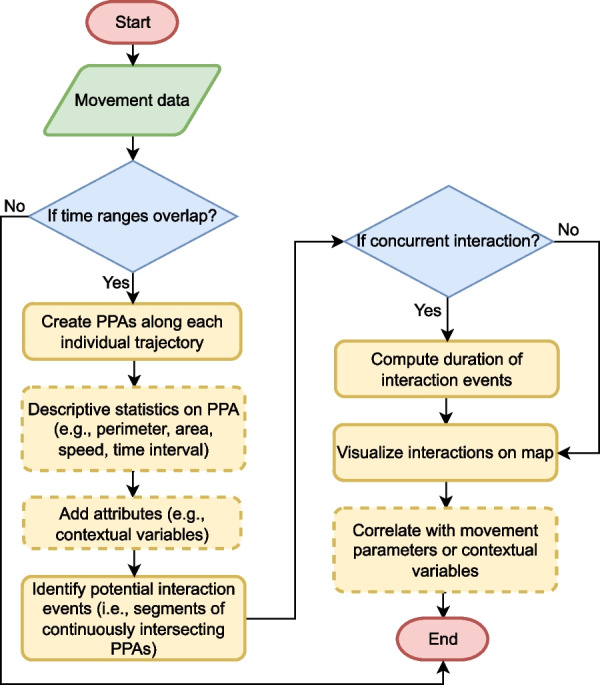
ORTEGA’s workflow for analyzing interactions between a pair of moving individuals using movement tracking data (dashed polygons indicate optional steps)

As illustrated in Fig. 3, ORTEGA first imports trajectories of two individuals (i.e. a dyad), as provided by the user. To conduct interaction analysis for more than two individuals, the user will need to employ *For* loops, by considering a moving entity as a reference at each loop and running the analysis in conjunction with all other individuals in the data set.

**Fig. 4:**
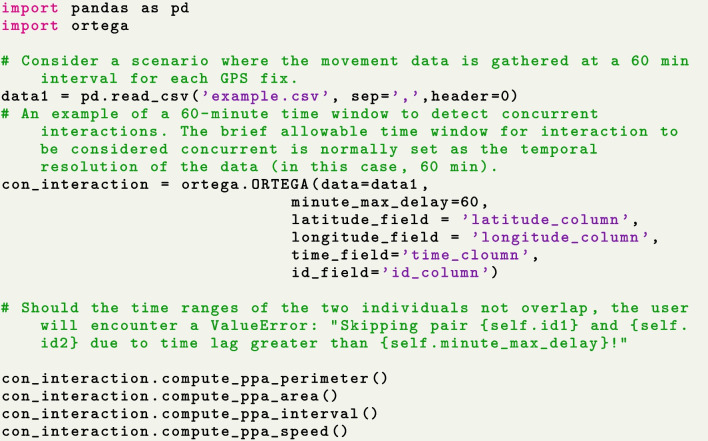
Initialize ORTEGA and conduct descriptive statistics

As shown in the below code snippet in Fig. 4, in the first step when initializing an ORTEGA object, the user is required to specify the column names for the identifier, longitude and latitude coordinates, as well as the timestamp. As previously mentioned in “ORTEGA’s algorithm description” section, the user is also required to define a brief time window denoted as $\tau$ to detect concurrent interactions, or a time interval $[\tau ^a,\tau ^b]$ to identify desired delayed interactions. As an example, the code snippet in Fig. 4 provides instructions on initializing ORTEGA to identify concurrent interaction. When the user provides a short time window (in minutes) as the value of the $minute\_max\_delay$ parameter (i.e., in this case, $minute\_max\_delay$ is considered as $\tau$), ORTEGA returns the results of potential concurrent interaction within that given brief time window. Alternatively, when both $minute\_min\_delay$ and $minute\_max\_delay$ are given (i.e., the user specifies a time interval $[\tau ^a,\tau ^b]$), ORTEGA detects delayed interactions that occur with a time lag within the specified interval (see the code in Fig. 6). Otherwise, the program will raise an error to remind the user to specify at least the parameter $minute\_max\_delay$.

During the initialization phase, ORTEGA will check whether the temporal spans of the two movement trajectories intersect. Should at least a partial overlap occur, the program will proceed to compute and generate PPAs along each individual’s trajectory; otherwise, it terminates with an error message. The output of the initialization phase is an instance of the ORTEGA class, which includes the original movement data and two lists containing the constructed PPAs of two moving individuals. Subsequently, the user can conduct descriptive statistics on PPA’s perimeter, area, speed, and time interval using ORTEGA’s descriptive statistics functions. Examples of implementing such statistics are shown in the same code snippet (see Fig. 4).

#### Identify concurrent interaction

**Fig. 5:**
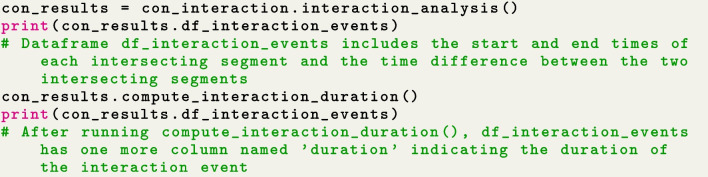
Implement concurrent interaction analysis

Next, the user can identify segments of continuously intersecting PPAs as potential interaction events using the function $interaction\_analysis()$, as demonstrated in the code snippet in Fig. 5. The outcome is a dataframe including the start and end times of each intersecting segment, along with the time difference between the two intersecting segments. The column of time difference indicates the time lag (if exists) between the two start points of the two intersecting trajectory segments. This information is especially helpful when the tracking of two individuals is not perfectly synchronized. It enables precisely pinpointing the location where the initial intersection or overlap of two trajectories occurred.

The user can use $compute\_interaction\_duration()$ subsequently to compute the duration of each interaction event, as presented in Fig. 5. Recall that in “ORTEGA’s algorithm description” section, it’s essential to consider the parameter $\delta _t$ for discerning between brief encounters and long-duration concurrent interactions. When the duration of an interaction event is shorter than $\delta _t$, it signifies an encounter, whereas a duration exceeding $\delta _t$ indicates a concurrent interaction. The outputs of interaction events are saved in a dataframe called $df\_interaction\_events$ as an attribute of $con\_results$. Each row in this dataframe indicates an interaction event. The user may take advantage of this dataframe to further interpret various types of identified interactions. In the provided example, where movement data is gathered every 60 min, a $\delta _t$ value of 60 min is sufficient to differentiate encounters from concurrent interactions.

#### Identify delayed interaction

**Fig. 6:**
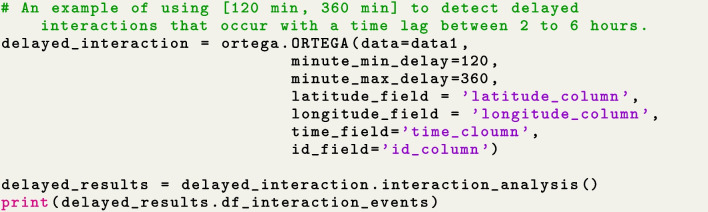
Initialize ORTEGA and conduct descriptive statistics

Figure 6 illustrates an example of identifying delayed interactions that occur with a time lag between 120 and 360 min. Similar to identifying concurrent interaction, the user needs to run $interaction\_analysis()$ after initializing an ORTEGA object. The outcome of this step is the same as the concurrent interaction, namely a dataframe called $df\_interaction\_events$ including the start and end times of each intersecting segment and the time difference between the two intersecting segments. In essence, the column of time difference in the case of delayed interaction shows the time lag of the delayed interaction event or the time lag between the two intersecting segments.

#### Contextualizing the identified interactions

Movement parameters such as speed and direction can be directly computed from GPS coordinates. Given a pair of consecutive GPS points, the speed of the corresponding PPA can be easily computed by dividing the Euclidean distance between the two given locations by the time interval. Subsequently, the *ratio of the difference in speed* (represented as $r_v$) between the two entities when potential interactions occur can be formalized as the absolute difference between the speeds of the two intersecting PPAs, divided by half of the sum of the speeds of the two intersecting PPAs. The value of $r_v$ ranges from 0 to 2, with 0 indicating identical speeds and a larger value indicating greater disparities in speeds between two interacting entities. Movement direction ranges from $-180$ to 180 degrees. *Similarity of movement direction* (represented as $r_\theta$) can be measured by the cosine value of the difference between the movement directions of the two intersecting PPAs [11]. This value equals 1 when movement segments have the same orientation, 0 when they are perpendicular, and $-1$ when they move in opposite directions. The ratio of the difference in speed and similarity of movement direction are automatically computed when running $interaction\_analysis()$. The user can further use this information to examine the correlation between movement parameters and potential interactions.

**Fig. 7:**
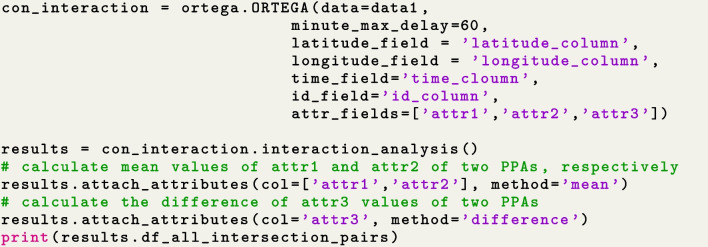
Contextualize identified interactions

In circumstances when contextual variables are available in addition to movement data, the user may leverage ORTEGA’s context-aware functionality to further inform interaction analysis. As Fig. 7 shows, the user may specify which contextual variables they want to include in the ORTEGA object during the initialization stage by specifying the $attr\_field$ parameter. Since each PPA consists of two GPS locations of an individual trajectory, each pair of intersecting PPAs includes contextual information of four GPS points of the corresponding PPAs of the two entities. ORTEGA offers a $attach\_attributes()$ function to aggregate these values at each potential interaction area (i.e. a PPA intersection). Currently, ORTEGA calculates the average of the values from the two GPS points that constitute each PPA. Subsequently, it computes either the average or the difference between the two mean values derived from the intersecting PPAs of the two individuals. This can be done by specifying the parameter *method* as ‘mean’ or ‘difference’. Ultimately, every pair of intersecting PPAs is associated with one value of a specific contextual variable. The user may correlate the identified potential interactions with movement parameters such as speed and movement direction, or with contextual factors. This correlation analysis can help us understand the relationships between potential interactions and individuals’ movement behaviors, as well as the surrounding environments where interactions occur. In the future extensions of ORTEGA package, the $attach\_attributes()$ function can be further advanced by incorporating more sophisticated methods, beyond mean and difference, to provide a more comprehensive and detailed representation of contexts around overlapping PPAs. Additionally, exploring other spatial relationships between contextualized PPAs in addition to ‘overlay’ can potentially offer a more comprehensive understanding of the influence of contexts on interactions between moving entities.

#### Mapping interactions

**Fig. 8:**
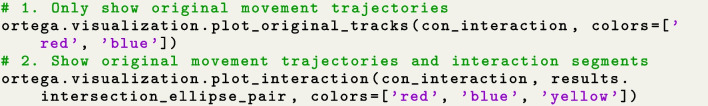
Mapping interaction events

Lastly, ORTEGA can visualize the detected interaction events and their associated intersecting PPAs alongside the original movement data for better interpretation. As shown in Fig. 8, the tool provides two visualization choices: (1) $plot\_original\_tracks()$: This option allows the user to visualize the original movement trajectories of both individuals, with PPAs depicted in red and blue (by default) for each individual, respectively. (2) $plot\_interaction()$: Selecting this option showcases the original trajectories while highlighting the overlapping segments in yellow by default, enhancing the clarity of the segments indicating potential interactions. The user may replace the parameter *colors* to customize the color code using common color names or hexadecimal color codes, especially if they wish to map the interaction between more than two individuals. Examples illustrating these visualization functions can be accessed at the provided example Jupyter notebook.^4^ Currently, the visualization offers a quick overview of the PPAs along the original movement trajectories of two moving individuals, with the intersecting PPAs highlighted.

## Results: case study

### Data sets

The goal of this case study is to demonstrate the functionality of ORTEGA v1.0 using GPS tracking data of two migratory turkey vultures (*Cathartes aura*). The tracking data, originally obtained from [27], has been continuously collected through 2021 [28, 29]. Hawk Mountain Sanctuary in Pennsylvania, USA, provides this data, which is available through Movebank Data Repository [17, 30].

**Fig. 9:**
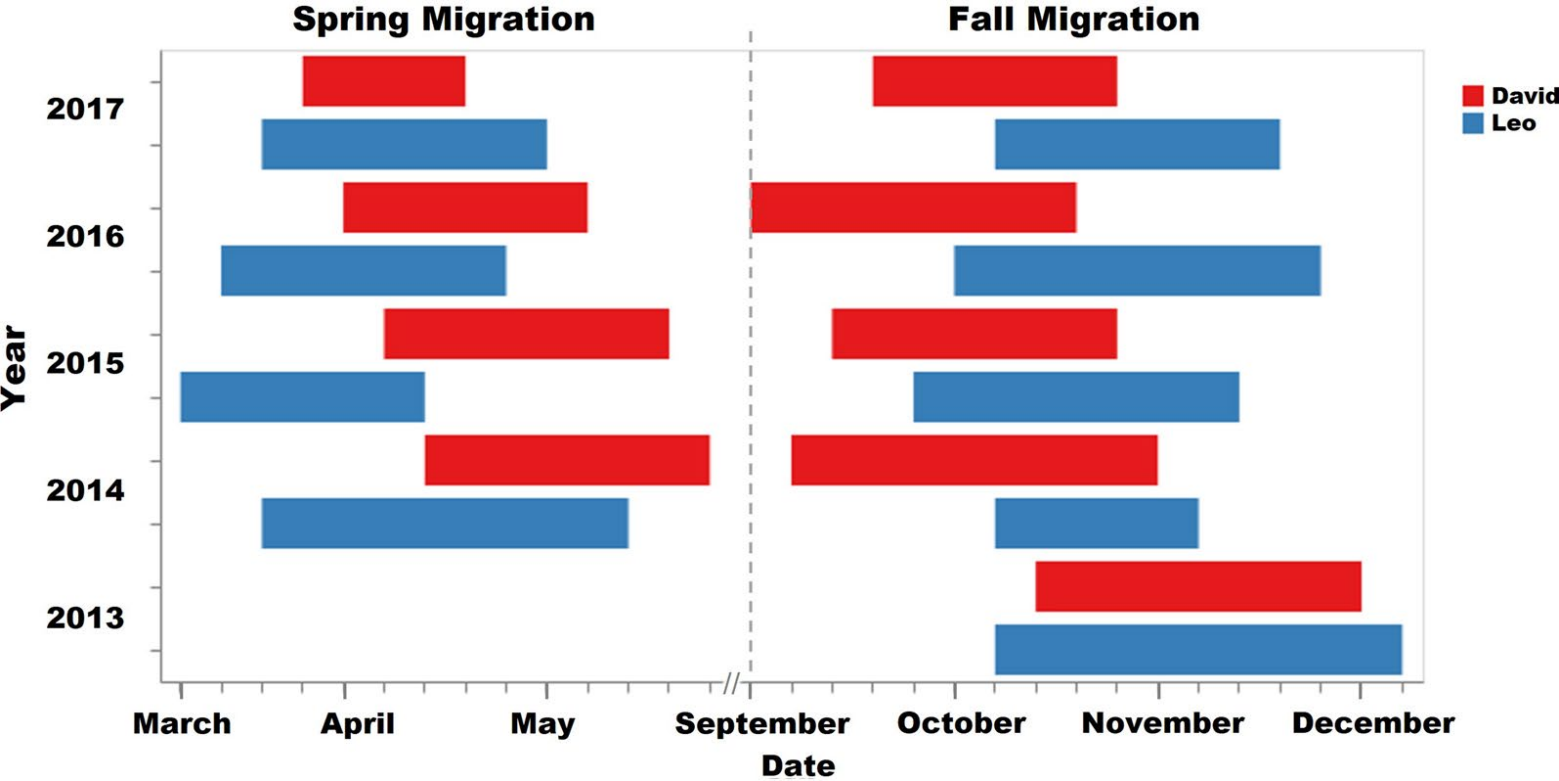
Spring and fall migration timeline of David in red and Leo in blue

Turkey vultures are North America’s most abundant obligate avian scavengers, boasting the broadest distribution. This case study focuses on two specific turkey vultures, “Leo” and “David”, both members of the interior North American population (*meridionalis*) [17]. This population is known for their migration from Canada to South America, across the central regions of North America. With a sampling rate of one hour per GPS fix, considering the low data quality of David’s tracking points after 2018, the common tracking period of the tracking dataset of David and Leo from 9 August 2013 to 31 December 2017, encompasses a total of 48,453 tracking points. Leo’s tracking data contain 22,615 points, of which 5037 points are during migration, and 17,578 points are during non-migration. David, on the other hand, covers 26,138 points in total, with 6,055 points captured during migration and 19,783 points during non-migration. The departure time of David and Leo during fall and spring migration each year (Fig. 9) are evaluated by mapping trajectories using DynamoVis [31].^5^

A corresponding step-by-step Jupyter Notebook with instructions for this case study is available on GitHub (See Footnote 4). The GitHub repository contains the movement data of the two migrant turkey vultures. Users can fully replicate the analysis demonstrated in this section.

### Descriptive statistics of movement parameter of PPA

We first illustrate the utility of ORTEGA with a case study between two migratory turkey vultures, “David” and “Leo” over the common observation period. After creating PPAs along their movement trajectories, descriptive statistics on the PPAs are computed, using the speed (meter per second) during migration and non-migration as an example.

**Fig. 10:**
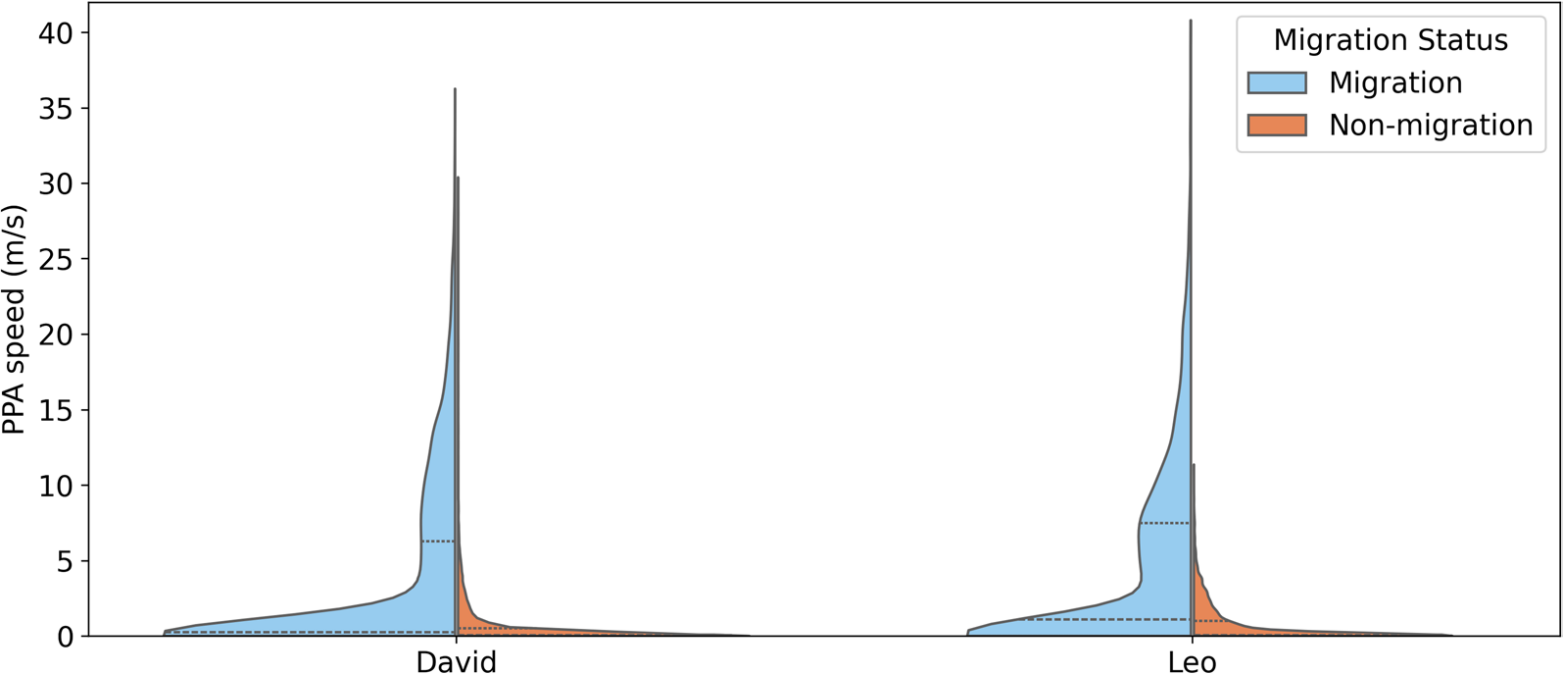
Distribution of PPA’s speed during migration (blue) and non-migration (orange) for David and Leo, both belonging to the same population. The dashed lines in each violin plot represents the first quartile, median, and the third quartile of the speed values

Figure 10 demonstrates a similar speed distribution of both vultures. Both birds displayed diverse movement behaviors, combining long-distance travels and more restricted movement (foraging and resting) during fall/spring migration. During migration, Leo averages approximately 4.5 m/s (std $\approx$ 6.2 m/s, median $\approx$ 1.1 m/s, maximum $\approx$ 40.8 m/s). David’s speed, on the other hand, averages around 3.7 m/s (std $\approx$ 5.7 m/s, median $\approx$ 0.3 m/s, maximum $\approx$ 36.3 m/s). During non-migration, Leo averages about 0.79 m/s, while David averages around 0.62 m/s. The broader speed spectrum, seen in both vultures, might point towards two dominant movement behaviors: instances of high-speed flight during migration, interspersed with slower movement or resting periods. Given that David and Leo usually undergo fall migration from September to November and spring migration from March to May [17], such patterns suggest high-speed flights during migration and low-speed movements or feeding sessions during non-migratory periods. In addition, Leo’s larger median and maximum speeds suggest it generally moves faster than David.

### Identifying concurrent and delayed interactions

Next, we demonstrate the utility of ORTEGA in identifying concurrent interactions between David and Leo between 9 August 2013 and 31 December 2017. Given that the sampling interval is one hour per GPS fix, we set the brief time window parameter $\tau$ at one hour to identify concurrent interactions. This experiment takes around two hours to complete on a Microsoft Windows computer equipped with a 2.5 GHz 8-Core Intel Core i9 processor and 64 GB RAM.

**Fig. 11:**
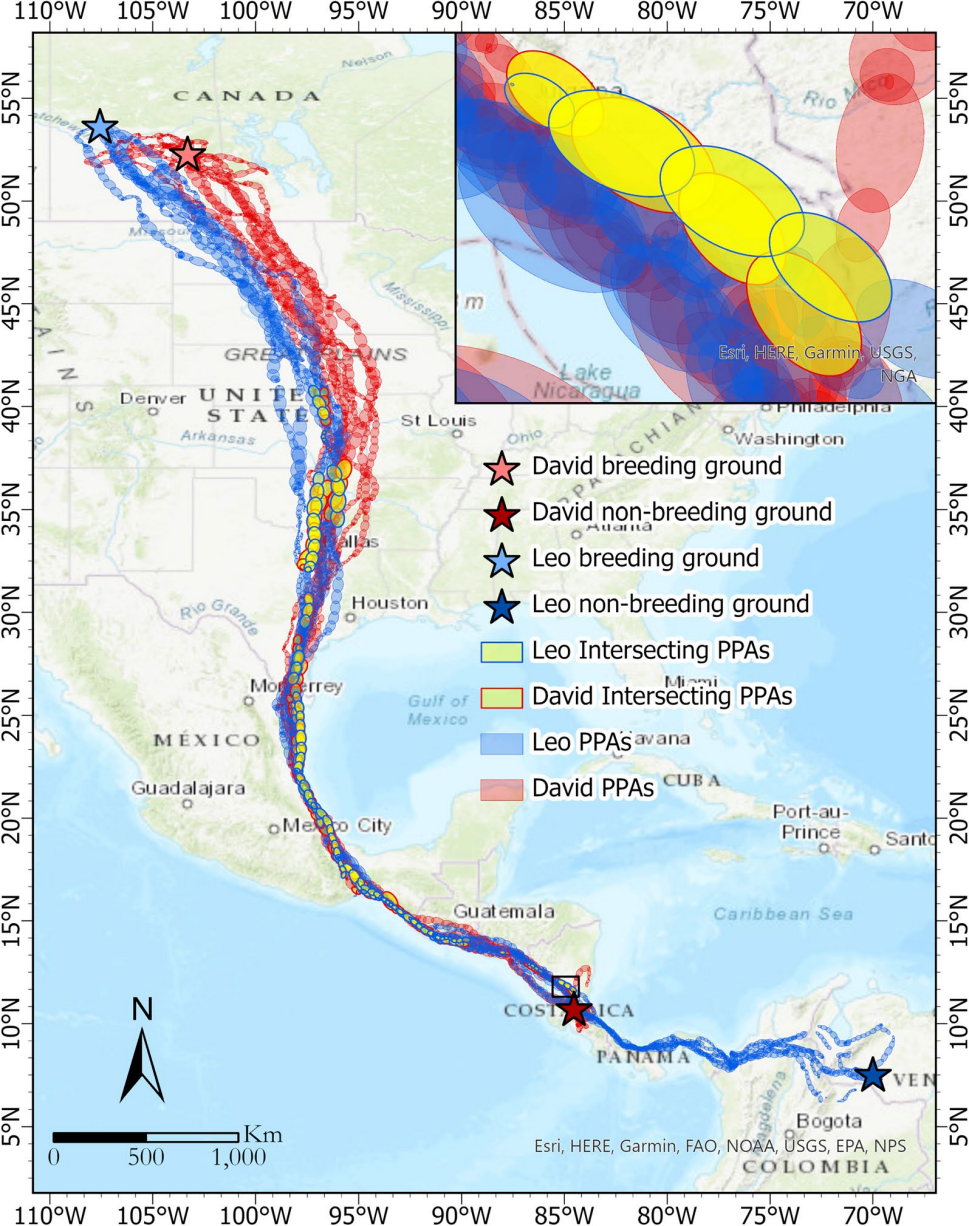
Identified concurrent interactions between David and Leo using ORTEGA. The PPAs of the two migratory vultures are shown using red (Leo) and blue (David) ellipses. The intersecting PPAs are highlighted in yellow, with borders colored to match the corresponding vulture’s PPAs. Breeding and non-breeding grounds of Leo are labeled as stars in light blue (coordinate: 53.7627°N, 107.5038°W) and dark blue (7.3158°N, 69.8679°W), respectively. Breeding and non-breeding grounds of David are in light red (52.2169°N, 103.1147°W) and dark red (10.0143°N, 84.0623°W). The inset map zooms into part of the intersecting PPAs. The map is generated using ESRI ArcGIS Pro Desktop 3.0.2

**Fig. 12:**
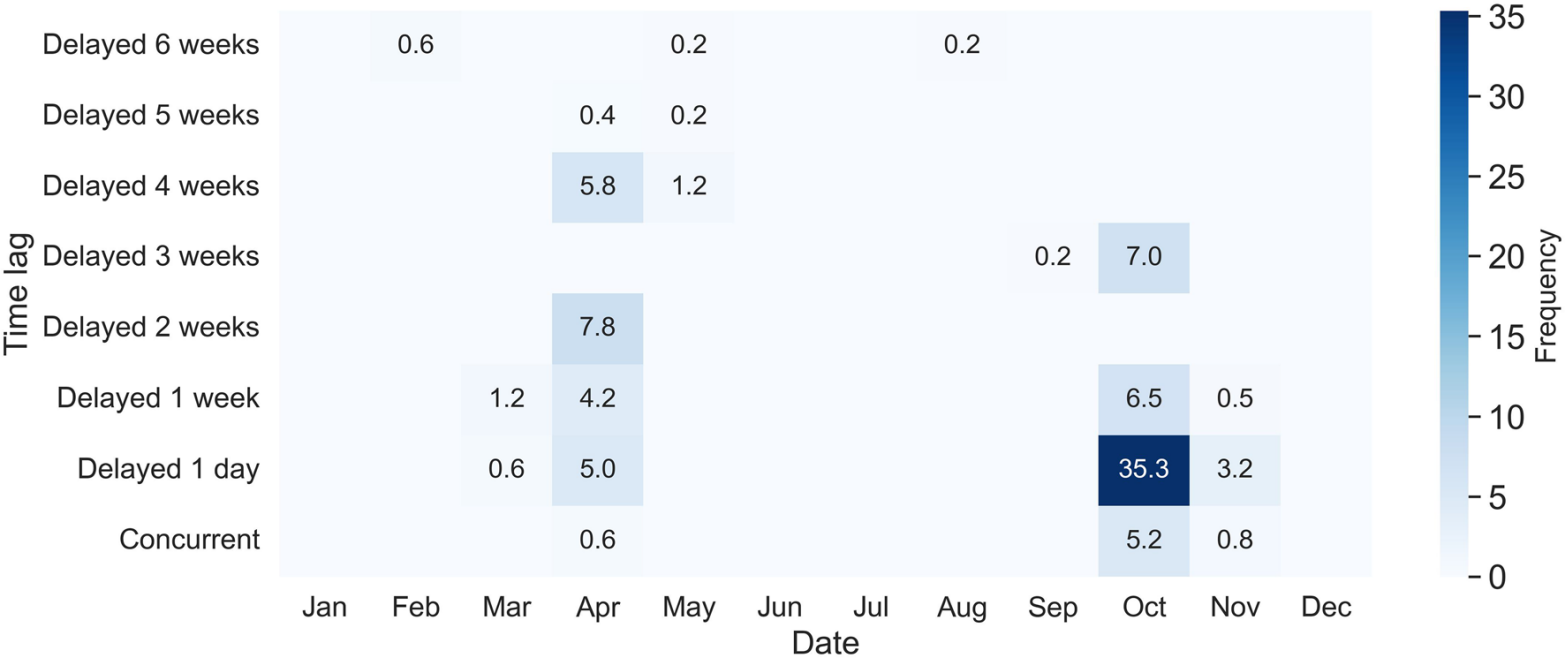
Average monthly frequency of identified concurrent and delayed interactions between David and Leo for a time lag from one hour to six weeks. Darker blue represents higher interaction frequencies

Figure 11 displays the GPS tracking data for these two migratory vultures (Leo in red and David in blue) from August 2013 to December 2017 with red and blue PPA ellipses, respectively. Locations of breeding and non-breeding grounds are labeled as stars. Potential concurrent interactions, indicated by intersecting PPAs, are highlighted with yellow ellipses. The sequence of intersecting PPAs identified around the Gulf of Mexico suggests closely aligned movement paths and the potential for joint flights by the two vultures. The highlighted intersecting PPAs can be used to trace where and when the two birds flew together along their migration. The average monthly frequency is summarized in the last row of Fig. 12. The monthly duration (hour) of each interaction event is calculated as well.

The results reveal 39 concurrent interaction events with an average duration of 5.2 h between these two migratory vultures over the five years. 80 percent of these migrations occurred in October. The outcomes show that the two birds are more likely to fly together during their fall migration as compared to the spring migration. Most of the interaction events seem to be located in the central parts of their migration path (latitudes between 13 and 41°N, longitudes between 97 and 90°W), suggesting that although the two birds might not start their migration together, they often catch up later along their migration paths as described below.

David and Leo, despite their distinct breeding and non-breeding grounds, exhibit interesting patterns of concurrent interaction during their migration seasons, particularly in the fall. While their non-breeding grounds are different, in Nicaragua for David and in Venezuela for Leo, both vultures migrate north to Canada for breeding (see Fig. 11). Leo’s breeding ground is located in the northeast of David’s. As seen on the map, overall, Leo’s migration journey is longer than David’s. Most of the concurrent interactions between David and Leo occur during the fall migration, with four out of the five detected events taking place in this season. Even though David typically starts his journey 7.8 days earlier than Leo on average, his frequent stops allow Leo to catch up. This synchronization predominantly happens in October or November around the Gulf of Mexico’s coast.

Notably, in 2015 and 2017, their paths intersected within the central US region (latitudes between 36 and 41°N). However, 2016 stands out as an exception: David’s swift and relatively uninterrupted migration seems to have prevented any joint flight that year. Leo’s consistent and faster journey to the same breeding ground each year implies that he may be more experienced in this migration route compared to David. In contrast, David’s initial wanderings, spanning two months in 2014 and approximately two weeks in 2015, before settling for a breeding ground, suggest his relative youth and inexperience. Documents from the field observations confirm that David was identified as a juvenile when he was captured for tagging in 2013 [28]. After 2015, David starts emulating Leo’s behavior by heading directly to a consistent breeding location. Leo’s spring migration departure is notably consistent, generally around 13 March. David, on the other hand, exhibits variability (from 17 April, 2014 to 29 March, 2017). While Leo sets off a month ahead of David in both 2014 and 2015, this difference narrowed to less than two weeks by 2017. Interestingly, 2017 is the only year when the two vultures show concurrent interactions during the spring migration.

Next, we demonstrate the application of ORTEGA in identifying delayed interactions between David and Leo. First, the time lag interval $[\tau ^a, \tau ^b]$ is decided by the departure time of David and Leo during fall and spring migration each year. The time lag intervals that are assessed for delayed interaction events include two hours to one day, one day to one week, one to two weeks, two to three weeks, three to four weeks, four to five weeks, and five to six weeks. A heat map showing the average monthly frequency of delayed interactions for different time lags is illustrated in Fig. 12. This can be interpreted as the average number of times within each month that one individual reaches the same location visited by another individual with a certain time lag, indicating a leader-follower behavior.

Figure 12 exhibits the average monthly frequency of identified concurrent and delayed interactions between David and Leo, which demonstrates a clear timeline of fall and spring migrations. October consistently exhibits higher interaction frequencies, which is a typical month for fall migrations, particularly for the delayed interaction with one-day, one-week, and two-week lag. In October, delayed interactions with one-day lag peak at an average monthly frequency of 35.3 h. April also shows moderate interaction frequencies, which is a typical month for spring migrations, particularly for delayed interactions with two-week and four-week lag. Other months like January, June, July, and December show minimal to no interactions across all delayed time lags as turkey vultures are located in separated breeding or non-breeding grounds in these months.

Delayed interactions with time lags of less than one week capture multiple migration events. Despite David’s earlier departures during the fall migrations of 2013 and 2014, we still capture David following Leo within one-week time lag. The delayed interactions with two and four-week time lags only capture the spring migration in 2016 and 2015, respectively, with David consistently departing 13–18 days later. The delayed interaction with a three-week time lag corresponds to the fall migration in 2016, with David departing 16 days earlier. Although no joint flights occurred during these specific migratory seasons, the presence of these delayed interactions provides compelling evidence of a shared migratory route between the two vultures.

### Incorporating contextual correlates in interaction analyses

To further contextualize the identified interactions using the movement of the two individuals, we use ORTEGA to assess movement speed and direction along the intersected PPAs. These additional variables are modeled as attributes of PPAs using ORTEGA’s context-aware functionality. Although we only use two movement parameters, ORTEGA is flexible to incorporate any movement and contextual variables as attributes of PPAs.

**Fig. 13:**
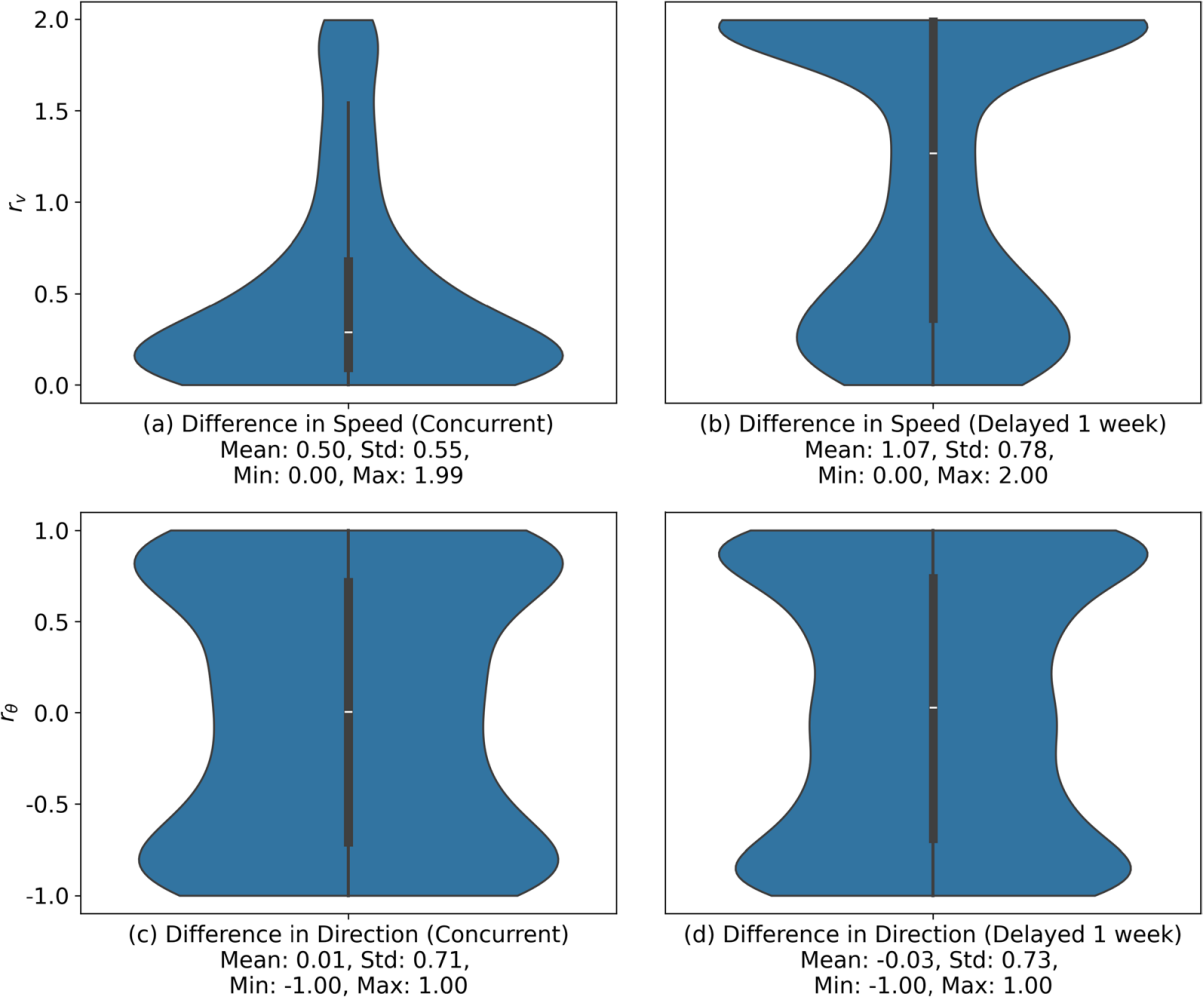
The ratio of the difference in movement speed during **a** concurrent and **b** delayed interactions and similarity of movement directions during **c** concurrent and **d** delayed interactions between intersecting PPAs of the two turkey vultures. The delayed interaction has a time lag interval from one day to one week

Figure 13 illustrates the distributions of the ratio of the difference in speed and similarity of the movement direction for the intersecting PPAs of the two vultures. It can be observed that the distributions of the ratio of the difference in speed values ($r_v$) show a skew towards 0 in concurrent interactions, suggesting similar speeds when flying together, and a skew towards 2 in delayed interactions, suggesting substantially different speeds when the two birds follow each other paths. As for the distributions of the similarity of movement direction ($r_\theta$), we observe peaks near 1 and $-1$ in both concurrent and delayed interactions, signifying similar and opposite directions, respectively. In concurrent interactions, similar movement speeds and similar movement directions are often observed when the two vultures migrate together, while opposite movement directions are more observed when the two vultures may forage or feed together. The larger speed differences and less skewness in movement direction differences detected in delayed interactions may suggest the presence of distinct migration and feeding patterns between the two vultures, separated by a time lag of up to one day. In general, the results underscore that vultures from the same population, when interacting concurrently, exhibit similar movement patterns and are influenced by comparable contextual factors, possibly indicative of joint flight migration.

## Conclusions

This application paper develops and evaluates a new open-source Python package for the analysis of dynamic interactions among moving entities, based on an extension of ORTEGA [6, 9]. Besides making the analytical code and example data set accessible to the community, the developed package also offers an extension of ORTEGA for incorporating context-aware abilities to inform interaction analysis. As a simple application example, using a case study of two migratory turkey vultures from the same population, we demonstrate the efficacy of ORTEGA in identifying and quantifying potential interactions for a flexible time lag between moving individuals during their migration. Two movement parameters, including speed and movement direction, are incorporated as the attributes of PPAs to further contextualize the identified interactions. The results indicate that in general, individuals belonging to the same population may show similar movement behaviors during concurrent interaction, which suggests potential collaborative or influenced behavior during their migratory patterns. Conversely, delayed interactions could indicate a more significant variance in terms of movement speed and direction. While only two birds are used in the case study, ORTEGA can be applied to analyze interaction among a group of individuals.

ORTEGA can be used to investigate dyadic, intraspecific, and interspecific interactions among moving animals. For example, [6] analyzed interspecific interaction between tigers and leopards using movement tracking data collected in Thailand, showing that tigers and leopards exhibit awareness of each other, and their interactions are primarily indirect and delayed. Additionally, using data from 67 tracked tigers, [32] identified four types of interactions: following, encounter, latency, and avoidance among tigers. ORTEGA was used to demonstrate how these behaviors are manifested in movements of interacting tigers and how their dynamics vary across gender and age. The newly added context-aware functions in ORTEGA opens up new possibilities for more sophisticated investigations into the relationship between contextual variables and potential interactions among moving animals. For instance, integrating variables such as land cover characteristics, vegetation, seasonality, and temperature into the context-aware interaction analysis model can enhance the ability to predict and contextualize interactions between moving individuals.

## Availability and requirements

Project name: ORTEGA

Project home page: https://github.com/move-ucsb/ORTEGA

Operating systems: Operating systems independent. The package was tested on Windows and MacOS.

Programming language: Python

Other requirements: Python 3.8 or higher.

License: MIT

Any restrictions to use by non-academics: None

## Data Availability

The tracking data of migrant turkey vultures are available on Movebank (https://www.movebank.org) for public usage at https://www.doi.org/10.5441/001/1.f3qt46r2.

## Abbreviations

ORTEGA: Object-oriented time-geographic analytical approach
PPA: Potential path area

## Footnotes

1 https://github.com/move-ucsb/ORTEGA.

2 https://pypi.org/project/ortega.

3 https://github.com/markvregel/STPtrajectories/.

4 https://github.com/move-ucsb/ORTEGA/blob/main/examples/example_turkey_vultures.ipynb.

5 https://github.com/move-ucsb/DynamoVis.
